# A Case of Ovariotomy

**Published:** 1870-09

**Authors:** M. A. Chase

**Affiliations:** Siruoza, Wisconsin


ARTICLE XXIV.

### A CASE OF OVARIOTOMY.

By M. A. CHASE, M.D , Siruoza, Wisconsin.

On July 4th, 1869, Miss M., aged 35, of sanguine, nervous temperament, consulted me in regard to a dropsical distension of her abdomen, and gave me the following history of her case:—

One year previous, while attending school, she noticed her abdomen was enlarging—did not know there was anything wrong with herself previous to this time; the distension commenced equally on both sides—no tumor or hardness in either side—was regular in menstruation.

She kept slowly filling up until the day I first saw her; Miss M. was looking pale and anemic, and had disease of the mitral valve.

I put her upon the use of digitalis, combined with a tonic, and an occasional hydroguge cathartic. Three weeks later, I visited her, and found the abdomen greatly distended, interfering with respiration, and patient unable to lie down. I introduced a trocar at the usual point, and drew off 15 quarts of pale, straw-colored fluid, of low specific gravity. A careful and thorough examination of the abdomen externally, did not reveal the presence of any tumor, and, previous to tapping, the patient, lying upon the back, gave the usual symptoms of ascites, fluctuation of the abdomen, and bulging at the sides. Continued my first prescription.

Eight weeks later, I visited the patient again, and found the abdomen distended as before; introduced the trocar, and drew off 14 quarts of dirty, greyish fluid, highly albuminous.

A careful examination of the abdomen at this time, showed the existence of a small tumor high up on the left side, under the cartilages of the ribs. Six weeks later, tapped again; fluid same color and amount as before. Tumor increased in size to nearly four times its size on discovery, and reaching down to the umbilicus, crescentic in shape, convexity looking to the left side, and concavity to median line of the body; the tumor had a peculiar hard feel. Drs. Tinker and Gatt saw the case with me, at this time. The tumor making its appearance in the position it did, and growing down into the abdomen was rather against its being ovarian. The patient, an old maid, would not permit a vaginal examination. The consultation threw but little light on the important subject of "what's the matter?" From this time to June 1st, 1870, the patient was tapped six times. At the last tapping, upon getting permission, I made an examination per vagina, and found the uterus drawn up so far I could not reach it. The solid part of the tumor had changed its shape, was nearly round, and fluctuated slightly. Came to the conclusion that the tumor was ovarian, told the patient so, and, as her health was failing, and she rapidly filled each time after tapping, I advised an operation. Her consent and that of friends having been obtained, the 11th day of July was set for the operation — the patient placed immediately upon the use of tr. ferri. gtt x. 3 times a day. On the 10th inst., I proceeded to the residence of my patient; found her in pretty good condition, and feeling hopeful. Ordered a mild purgative.

On the morning of the 11th, assisted by Drs. Tinker and Gatt, I proceded to operate, the patient's bowels having been freely moved the night before. The urine was drawn off; I introduced the trocar and drew off 20 ℔s. of highly albuminous fluid of a dirty greyish color. The tumor extended from under the ribs, down below the umbilicus. Gave Morph. sul. gr. *ss*, in half ounce brandy. The patient was placed upon the table, and the anæsthesia commenced with a mixture of one part chloroform and two parts ether. After spending some time with this, and the patient not coming fully under the influence of it, one drachm of pure chloroform was used, producing full anæsthesia. Temperature of room, 77° F. Everything being in readiness, I made an incision on the median line, midway between the umbilicus and pubis, including the wound of the trocar, down to the peritoneum, making a small opening through this membrane, I introduced a probe pointed bistoury, and enlarged the opening each way to the extent of about an inch and one-half; introducing my finger I found extensive adhesions. I enlarged the opening upward about four inches, making an opening seven inches in length. Introducing my hand, I proceeded, as gently as possible, to break up the adhesions by tearing the fibrous bands from the surface of the tumor. Some were very firm, others not so firm. Brought forward a cyst the size of a quart bowl; introduced the trocar and evacuated the contents.

Passing my hand up on the left lateral and anterior surface of the tumor, I found the adhesions very firm, the walls of the tumor and peritoneum adhering firmly together over a large surface. I broke up the adhesions at this point by slowly working my fingers between the peritoneum and tumor. Considerable force had to be used. Passing my hand under the tumor it came in contact with fibrous bands connecting the intestines and tumor; these were torn from the surface of the tumor. The tumor being free, was lifted from its bed in the intestines; the intestines were covered with soft cloths wet in warm water. The pedicle was thin, about 3½ inches broad, and supplied with large bloodvessels.

It was tied with a ligature made of saddler's silk doubled —a needle armed with a double ligature of this was passed through the centre of the pedicle, close to the tumor, and tied both ways with three knots. The pedicle was then separated from the tumor with the scissors, the ligatures were cut short, and the stump returned to the abdomen. Some blood among the intestines was removed by a very soft cloth, wet in warm water, and laid on the intestines to absorb the blood.

The wound was closed by four silver sutures, carried *through* the peritoneum, and long strips of adhesive plaster placed between the sutures. Two narrow compresses, wet in warm water, were placed one on each side of the incision, and a broad flannel roller passed around the body. Patient placed on her bed with shoulders and feet elevated.

Operation ended at 12.20. Tumor multilocular, weighing 35℔s. Pulse 90; gave morph. sul. gr. 1. 12.35 P.M. Patient complains of great pain in abdomen; gave 20 gtt. tr. opii. 1 P.M. Extremities cool; ordered warm dry flannels; pain continues—½ gr. morph. sul. 3 P.M. ½ gr. morph. sul. Pulse 100. 4 P.M. Patient inclined to dose.

6 P.M. Pulse 108; ¼ gr. morph. sul. Examined wound and found but little oozing. Patient allowed small pieces of ice in the mouth and iced milk. 8 P.M. ¼ gr. morph. sul.; patient comfortable.

July 12th—6 A.M. Passed catheter; patient slept some. Had ¼ gr. morph. sul. every four hours. Pulse 100—good; examined wound—found but little oozing. 9 A.M. Pulse 93, soft; ¼ gr. morph. sul. 10 A.M. Patient vomited a little curdled milk; oozing of bloody serum from lower angle of wound. 1 P.M. Vomited a little; pulse 90, good. 6 P.M. Pulse 100; tongue dry in the centre, and skin dry; cheeks slightly flushed. Ordered ½ gtt. fl. ex. aconite rt. every hour. 7 P.M. Wound looking well; slight tympanitis of abdomen—not tender—free from pain—catheter. Respiration 20; pulse 112. 10 P.M. Pulse 98, soft; tongue not as dry; aconite to be given every two hours; allowed ice *ad libitum.* 12 M. Pulse 100, easy.

July 13th—6 A.M. Slept some. Pulse 100, soft. Tongue moist—catheter. 12 M. Some tenderness in abdomen; ¼ gr. morph. sul. 8 P.M. Pulse 108, soft; catheter. July 14th. Slept nearly all night; pulse 100; wound looking well; catheter. Slight distension of abdomen from gas, for 48 hours past, leaving. The respiration has been good at all times. 6 P.M. Pulse 96; wound looking well; bowels feel like moving off; gave injection of strong soapsuds, and brought away a large quantity of feces. July 15th—6 A.M. Some pain in back; ¼ gr. morph. sul. Allowed mutton broth. July 18th. Pulse 70; removed sutures; some suppuration around sutures; wound healed by first intention.

21st. Patient doing well.

27th. Patient up about the house.

August 12th. Patient at work about the house, feeling as well as ever, and gaining flesh rapidly.

